# Epigenetic Codes Programing Class Switch Recombination

**DOI:** 10.3389/fimmu.2015.00405

**Published:** 2015-09-11

**Authors:** Bharat Vaidyanathan, Jayanta Chaudhuri

**Affiliations:** ^1^Immunology and Microbial Pathogenesis Program, Weill Cornell Graduate School, New York, NY, USA; ^2^Immunology Program, Sloan Kettering Institute, New York, NY, USA

**Keywords:** AID, DNA repair, DNA deamination, recombination, non-coding RNAs

## Abstract

Class switch recombination imparts B cells with a fitness-associated adaptive ­advantage during a humoral immune response by using a precision-tailored DNA excision and ligation process to swap the default constant region gene of the antibody with a new one that has unique effector functions. This secondary diversification of the antibody repertoire is a hallmark of the adaptability of B cells when confronted with environmental and pathogenic challenges. Given that the nucleotide sequence of genes during class switching remains unchanged (genetic constraints), it is logical and necessary therefore, to integrate the adaptability of B cells to an epigenetic state, which is dynamic and can be heritably modulated before, after, or even during an antibody-dependent immune response. Epigenetic regulation encompasses heritable changes that affect function (phenotype) without altering the sequence information embedded in a gene, and include histone, DNA and RNA modifications. Here, we review current literature on how B cells use an epigenetic code language as a means to ensure antibody plasticity in light of pathogenic insults.

## Introduction

Genes are the basic molecular unit of heredity in all organisms since it is the gene (genotype) and not the trait (phenotype; except for imprinted genes) that is inherited. A gene usually refers to a particular sequence of nucleotides that has an annotated function. However, phenotypic manifestations can be governed by factors beyond (epi) genes (genetics), which too are hereditary. Epigenetic alterations are carried out by a repertoire of modifiers (writers and erasers) and readers, which can act on either proteins (histones) or nucleic acid (DNA/RNA) (1–3). Epigenetic regulation also includes non-coding RNAs (micro-RNA and long non-coding RNA), which can directly or indirectly (via recruitment of proteins) affect gene expression (4–6). This complex layer of gene regulation is a testament to the pliability that the system needs and possesses. The immune system exemplifies one such complex system that is geared to adapt to the environment, and B cells that make antibodies also need to reshape their antibody repertoire during antigenic challenges. Thus, it is not surprising that B cells overcome genetic constraints and integrate environmental cues into a complex network of gene regulation, which is both flexible and heritable. Dynamic epigenetic alterations engineered with spatiotemporal precision that expand the genetic code beyond A, G, C, and T, would be ideal for B cells in their quest to diversify the antibody repertoire during infection by an ever-evolving array of pathogens.

**Figure 1:**
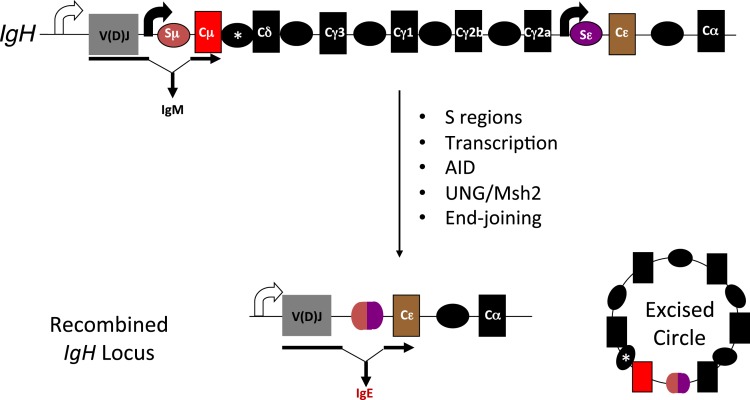
**Overview of CSR**. Each of the CH genes is preceded by transcribed, repetitive DNA elements called switch (S) regions. AID mediated DNA deamination, followed by processing of deaminated cytidines by components of the base excision and mismatch repair machineries (UNG and Msh2, respectively) lead to the generation of DNA double-strand breaks. End-joining of ­double-strand breaks between donor and acceptor S regions completes CSR. Scheme for CSR to IgE is shown; block curved arrows indicate germline transcription from promoters upstream of Sμ and Sε; *represents a putative, but uncharacterized switch region upstream of Cδ.

Class switch recombination (CSR) is one such secondary antibody diversification that occurs in peripheral lymphoid organs when B cells encounter antigen, and is dependent on cytokine/chemokine cues generated by T cells and stromal cells (7). The switching of the antibody isotype from IgM (or IgD) to IgG, IgE, or IgA is necessary to impart distinct effector functions (8, 9). At the molecular level, CSR is a deletional-recombination reaction occurring between repetitive DNA elements, called switch (S) regions that precede each constant region (C_H_) segment. Cytokine stimulation and/or antigen binding to B cells stimulate “germline” transcription through the S regions and promote accessibility of the DNA deaminase AID (activation-induced cytidine deaminase), whose activity leads to the generation of DNA double-strand breaks (DSBs) at S regions. End-joining of DSBs between donor (usually Sμ) and acceptor S (Sγ, Sε, or Sα) regions replaces Cμ for a different C_H_ gene segment downstream of the rearranged variable region segment to complete CSR (Figure 1). The drivers of germline transcription, AID expression, its target specificity and factors in end-joining have been extensively studied and reviewed elsewhere in Ref. (8, 10–13).

The role of histone modifications, DNA methylation, and non-coding RNAs during CSR has been recognized and appreciated significantly in the last decade (4, 14, 15). Given that B cells encounter an inflammatory milieu during an immune response and since the same cues can also program epigenetic changes, it suffices to say that there must be an underlying intricate chromatin network landscape, which is shaped to govern CSR. In this review, we will focus on recent advances that buttress the existence of epigenetic codes that orchestrate CSR.

## Epigenetic Control of Switch (S) Region Locus Accessibility and Antibody Response

S regions are highly repetitive G-rich sequences that precede each constant region gene. Transcription through these regions is necessary for CSR. However, constitutive (donor S region) and inducible (acceptor S region) transcription is associated either with a poised and dynamic epigenetic state, respectively (4). Donor S region (Sμ) has a transcriptionally active state even in naïve B cells, suggesting that it is poised for participation in CSR. Histone modifications like H3K4me3 and H3K9ac are present at Sμ in naïve B cells and increases upon stimulation (16–19). These are marks associated with an open chromatin conformation that would favor accessibility of the CSR machinery including tethering AID to donor S region.

**Figure 2:**
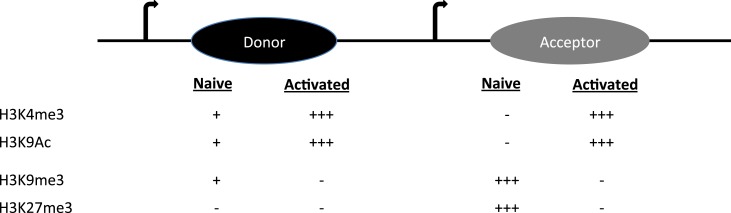
**The histone modifications on donor and acceptor S regions in naïve and activated B cells are shown**.

On the other hand, the acceptor S regions (Sγ1, Sγ3, Sγ2a, Sε, and Sα) are inaccessible to the CSR machinery at the basal state. When the B cell faces an antigenic challenge that provides CSR triggers including T cell help (CD40 ligation), pattern-recognition receptor ligation and the cytokine milieu (IL4, TGFβ, retinoic acid, BAFF, and IFNγ), the cognate S acceptor region accumulate histone modifications that are permissive to transcription and accessibility of only the particular S acceptor participating in CSR. The alterations include removal of repressive chromatin marks (H3K9me3 and H3K27me3) and also induction of H3K9ac and H3K4me3 (Figure 2) (17, 18). Knockdown of histone chaperone FACT complex components (SSRP1 and SPT16), Spt6, and methyl transferases (Ash2 and Wdr5) in CH12F3 cells have revealed the pertinence of the histone marks in regulating locus accessibility and S region cleavage, and thus CSR to IgA, a function which extends beyond germline transcript induction (20–22). If the same holds true in primary B cells and is it generalizable for CSR to other antibody isotypes remains to be determined. Additionally, H3K9me3 mark found at donor S region can recruit HP1γ-KAP1 complex, which facilitates AID tethering (17). The enzyme Suv39h1, which is probably the histone methyl transferase involved in the deposition of this histone mark, plays a positive role in inducing CSR to IgA, since deletion of the gene impairs IgA CSR in primary B cells without affecting GLT (23). It is not specifically clear as to why there is isotype specificity if H3K9me3 is a donor S region mark.

A word of caution in the interpretation of all these results is warranted since most of the studies show strong correlative but not direct causative evidence; genetic models targeting the histone modifying enzymes would be required to unequivocally support the claims. Even the use of genetic or pharmacologic approach to manipulate histone modifications might not provide conclusive proof of their specific role in locus accessibility due to functional promiscuity/essentiality of the enzymes. Future studies are necessary to uncover the complex relationships that probably exist between multiple factors influenced by a single histone modification, and how an ensemble of spatiotemporal histone modifications orchestrates CSR.

The function of mixed-lineage leukemia (MLL)-like H3K4 methyl transferase complex in CSR was tested by deletion of a key component PTIP conditionally in B cells (24, 25). These studies revealed that compromised H3K4me and acetylation and global chromatin architectural changes associated with the loss of PTIP leads to decreased S region accessibility, germline transcription, and thus compromised CSR to multiple isotypes (24, 25).

The function of histone acetylation/deacetylation in B cells was uncovered by conditionally inactivating MOZ (H3K9 acetyl transferase) and HDAC-1 and -2 genes in mature B cells (26, 27). Stage-specific MOZ deletion suggested that it is required for optimal GC response (proper affinity maturation and memory formation) (26, 28). HDAC-1/2 were absolutely necessary for the proliferative burst that B cells undergo during stimulation for CSR, and double-deficient B cells fail to divide and die by apoptosis when challenged with LPS + IL4 (29). In contrast, pharmacological intervention with HDAC inhibitors, butyrate and valproic acid, *in vivo* during T-dependent/independent antibody response and *ex vivo*, did not affect B cell proliferation and viability (30). However, they did block CSR and plasma cell differentiation by upregulating cognate micro-RNAs that dampen AID and Blimp1, critical for CSR and plasma cell differentiation, respectively. Interestingly, the therapeutic potential of HDAC inhibitors in treating antibody-mediated lupus was highlighted by the finding that they ameliorated disease and prolonged survival in a mouse model of lupus (30). Discrepancies between genetic and pharmacological approaches can be due to milder effect of inhibitors or more broad effects on multiple HDACs beside HDAC-1/2.

Finally, the function of polycomb repressive complex (PRC)-2 component, Ezh2 (histone methyl transferase), which was shown to be necessary for B cell development (31), during GC response was tested by conditional inactivation of Ezh2 using Cγ1-cre (32, 33). Two independent studies provided compelling evidence that the germinal center (GC) formation and consequent cell-fate decisions including CSR, SHM, plasma cell differentiation and memory response are critically shaped by Ezh2 (32, 33). H3K27me3 and H3K4me3 ChIP-Seq in Ezh2-sufficient versus -deficient GC B cells revealed that Ezh2 negatively regulates terminal differentiation by epigenetically (via PRC2) regulating Blimp1, Xbp1, and Irf4, and thus promotes long-lasting immunity by sustaining antibody diversification and memory B cell differentiation (26, 33). Gain of function mutant Ezh2 alleles (found in lymphoma patients) cause GC hyperplasia in mice and cooperate with Bcl2 to accelerate and sustain malignant transformation of GC B cells (32). Combinational therapeutic targeting of Ezh2 for specific B cell malignancies (GC-DLBCL) will definitely be enticing (32, 33).

## Epigenetic Control of AID Expression

AID is the key enzyme that is essential for CSR. It is probably one of the only proteins unique to stimulated mature B cells, and AID fate-mapping studies corroborate that physiologically functional levels of expression are largely restricted to B cells (34). It instigates DNA lesions in the form of deaminated deoxycytidine (dC), i.e., deoxyuridine (dU) at donor and acceptor S regions, which is subsequently processed by the general base excision repair, mismatch repair machineries, and DNA end-processing enzymes, such as, Mre11 and CtIP to generate DNA DSBs (10). Since it can induce DSBs, it is a potential mutator, and thus, AID expression is stringently controlled at the transcriptional and post-translational levels (8, 12). However, herein we review epigenetic factors modulating AID expression.

At the epigenetic level, AID expression is controlled by DNA methylation–demethylation and micro-RNAs. The AID locus, especially the promoter sites for cognate stimuli-induced transcriptional factors are hypermethylated in naïve B cells and the mark is reversibly modulated during different stages of B cell-fate program (35). Activated B cells or GC (GL7+Fas+) B cells acquire a permissive epigenetic landscape of hypomethylation that allows robust AID expression by STAT6, NF-κβ and Hox-C4 transcription factors (4). Besides, phylogenetic footprinting, histone acetylation, and DNase1 hypersensitivity site (DHS)-mapping revealed that the AID locus is dynamically shaped during an ongoing immune response (36). Histone H3 acetylation is increased at AID regulatory regions upon *in vitro* stimulation of naïve B cells and in GC B cells. In activated B cells, a conserved non-coding sequence 7kb downstream of the AID locus maps to a DHS and regulates AID expression positively, via the binding of a yet to be identified protein (36). However, AID is turned off epigenetically upon terminal differentiation, probably as a means to preserve antigen specificity of the antibody secreting B cells (36).

Post-transcriptional regulation of AID by micro-RNAs 155, 181b, 93, and 361, provides an additional layer of safeguard against a potent genome mutator (12). miR155, is the best studied one, which suppresses *aicda* expression by binding to a canonical site on the 3′-UTR of *aicda*. Although miR155 has functions way beyond suppressing AID in B cells undergoing CSR, as evidenced by compromised CSR in absence of miR155, yet its specific effect on regulating AID is significant. Mutation of the miR155-binding site on *aicda* 3′-UTR leads to increased AID levels that potentiate cMyc-IgH translocations (37, 38). Besides, miR155 and AID levels are inversely correlated in Burkitt’s lymphoma, and an IL10/miR155 axis can potentially modulate AID expression during chronic inflammation and lymphomagenesis (39).

## Epigenetic Control of AID Targeting

An enzyme like AID is a dual-edged sword; on one hand, it is mandatory for optimal humoral immunity but on the other, a threat to genomic integrity. Therefore, a normal B cell must delegate adequate layers of safeguard in addition to regulating AID expression, which would primarily target AID to the physiological targets. Genetic factors controlling AID targeting and function have been reviewed elsewhere in Ref. (12, 13, 40). Herein, we will focus on epigenetic guides of this potent mutator.

### Histone modifications

One way to limit the risk of collateral damage would be to sequester AID at hotspot target motifs. S regions are GC-rich and possess stretches of 5′-AGCT-3′, which are AID hotspots (13). These regions, when transcribed form stable R-loop structures that provide single-stranded DNA substrates for AID (10). An intriguing finding is that histone modifications, such as, H3S10 phosphorylation induced in CSR-activated B cells have also been linked to R-loop formation (41). Stable R-loops formed during CSR stimulation of B cells at S regions also accumulate H3K9AcS10ph modification. The classical adaptor protein 14-3-3, which has unique specificity for 5′-AGCT-3′ repeats and H3K9AcS10ph modification, also directly binds AID (42, 43). Thus, it is well poised to recruit AID to recombining S regions during CSR, thereby serving as transducers of the epigenetic code (4). It remains to be seen, however, if genome-wide occupancy of 14-3-3, H3K9AcS10ph, and AID overlap, or if 14-3-3 only functions during physiological AID targeting. Another study focused on the chromatin-bound AID-interactome to reveal that the RNA polymerase-associated factor (PAF) complex member LEO1 is required for efficient targeting of AID to Sμ in CH12F3A cells (44). It will be interesting to test if the function of LEO1 is also pertinent in B cells undergoing *ex vivo* CSR and more importantly in GC B cells. AID was also shown to bind the KAP1-HP1γ complex, the latter of which recognizes H3K9me3 (17). AID targeting was dependent on KAP1 and also on its association with HP1, since genetic manipulation studies clearly revealed that AID occupancy at Sμ was dampened due to loss of KAP1 alone or its interaction with HP1 (17).

### Super-enhancers and regulatory clusters

Enhancers are classically defined as a class of DNA elements that function in promoting transcription of gene from a distance, and irrespective of their orientation with respect to the target gene (45). Advance is sequencing techniques like DHS mapping (Dnase-seq), ChIP-seq and 3C-5C, Hi-Seq in the last decades has enabled genome-wide characterization of enhancers. Key features include presence of Dnase1 hypersensitive sites, multiple transcription factor binding sites, histone modifications like H3K4me1 and H3K27Ac, and looping to contact promoter elements far apart in the genome (46). Essentially now, the presence of a chromatin profile as alluded to above is considered as a hallmark of enhancers, although to date these are strongly correlative yet not always functionally causative. A new class of regulatory DNA elements has been defined recently, termed super-enhancers or stretch-enhancers (47, 48). These are cell-type specific enhancers that play a key role in establishing lineage or cell identity (47, 48). They are defined as clusters of large regulatory domains that have remarkable enrichment for transcription factor and coactivator (Mediator) binding along with a characteristic chromatin landscape (nucleosome occupancy and histone modifications) (46). Since AID expression and CSR are unique to B cells, it would make sense to regulate this cell-type specific expression and targeting by integrating stimulation cues to topologically associated domains i.e., super-enhancers. Recent work from several laboratories has greatly advanced our understanding of AID targeting/mistargeting (49–51).

**Figure 3:**
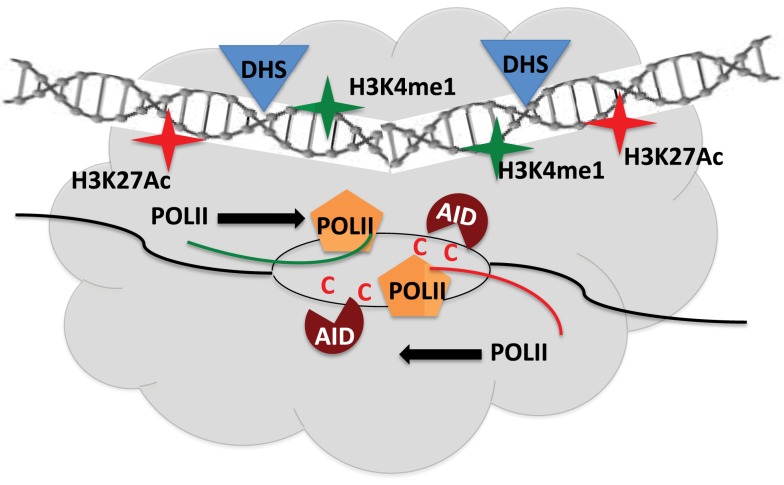
**Regulatory clusters containing super-enhancers modulate AID mistargeting**. Convergent transcription between promoters of target gene and super-enhancers from topologically associated domains (TAD) allow access of AID to single-stranded DNA and promote off-target activity. DHS – Dnase1 hypersensitive sites.

Using genome-wide sequencing approaches including GRO-seq, DNase-seq, and ChIP-seq, AID targets were found to be mostly unique in different cell-types (B cells, MEFs) although they shared common features of being transcriptionally active regions. However, transcription alone was not sufficient to explain the distinct set of hotspot in MEF versus B cells (49). Analysis of genes transcribed in both cell-types but only targeted in one, led to identification of a shared set of epigenetic attributes including H3K27Ac and H3K36me3, which typify enhancers (49). Deep sequencing techniques allowed uncovering a remarkable overlap of AID target sites to regions of the genome that constitute super-enhancers (50, 51). The AID off-targets like Cd83 in CSR-activated B cells map to regions enriched in chromatin marks, typical of super-enhancers, and lie within sites of convergent transcription (sense transcription from promoter of genes and anti-sense transcription from super-enhancer) (50, 51). A majority of AID-instigated lesions (irrespective of cell-type) occurred at transcription start sites, which were connected over long distances to multiple topologically active “regulatory clusters” (51). A central theme that came out from these elegant studies is that the nuclear microenvironment, which can vary from one cell type to another (even between *ex vivo* CSR-activated B cells and GC B cells), greatly influences AID target selection, yet the targets do share the following commonalities: (a) highly transcribed super-enhancers, (b) topologically interconnected clusters, and (c) sites of convergent transcription (50–52) (Figure 3). How these findings fit the available models of stalled Pol-II (Spt5), 14-3-3, RNA exosome, PTBP2-mediated AID targeting remains to be explored (8). Additionally, these strong correlative evidences should now be tested for causation using the CRISPR-Cas9 system by abrogating transcription or knocking out the eRNA transcripts, to query if it affects the mutational landscape in AID-expressing cells. Another open question is whether convergent transcription regulates physiological Ig locus targeting of AID during CSR. These exciting avenues remain to be explored and would be at the forefront of research of AID biology in the coming years.

### Non-coding RNA

AID, being a member of the APOBEC-family of enzymes, has long been known to be associated with RNA and RNA metabolism-associated factors, such as, RNA Pol-II, Spt5, RNA exosome, PTBP2, and CTNNBL1 (8). Since AID activity has been strongly linked to transcription, R-loop formation and anti-sense RNA processing, a recent study delved into the details of how non-coding RNA biology can impact AID targeting (14). Using a mouse model of conditional inactivation of an essential component of the RNA exosome (Exosc3), it was revealed that the RNA exosome shapes the non-coding transcriptome in B cells in a way that allows AID to access sites of anti-sense and divergent transcription (14). This was true for many of the well-characterized off-target sites of AID in B cells including Cd79b, Cd83, Pim1, IL4ra, and cMyc. However, the proposed model of divergently transcribed loci generating RNA exosome substrates facilitating single-stranded DNA access to AID is in contradiction with the convergent transcription model (14, 50), and future work is necessary to address the discrepancies.

Another elegant finding of Exosc3 and 10 conditional deletions in mature B cells was that it unraveled a novel role of this cellular RNA degradation factory in regulating enhancer (e) and super-enhancer (se) RNAs, which as discussed before might have a remarkable impact on AID mistargeting (14, 50, 51, 53). The genome-wide mapping of changes in non-coding RNA transcriptome in RNA exosome-deficient B cells undergoing CSR led to the identification of a distal divergent eRNA-transcribing element (lncRNA-CSR) (53). Ablation of transcription from this element profoundly impacted looping-dependent long-range DNA interactions with IgH 3′ regulatory region super-enhancer and compromised CSR. Additionally, the RNA exosome appears to promote genomic integrity in activated B cells by chewing up genome-destabilizing R-loop structures that emanate from active enhancers and also by regulating chromatin silencing (53). Questions come to mind as to how the CSR-promoting R-loop structures at S regions are preserved temporally, and how the RNA exosome function in facilitating template strand deamination by AID is in unison with its more global functions in genomic integrity (54). Nonetheless, these compelling studies have definitely united super-enhancer and ncRNA biology into B cell research, especially CSR, and will propel the field forward in coming years.

Co-transcriptional processes and factors including RNA pol-II stalling, RNA exosomes, and even eRNA/seRNA appear to reinforce the transcription and R-loop dependent AID targeting model (12, 14, 16, 50, 51, 53, 54). It can be concluded that the requirement for transcription (convergent/divergent) for AID targeting/mistargeting has been testified. However, an intriguing finding predating the discovery of AID was that processing of switch transcripts by splicing was also necessary for CSR (55, 56). The role of transcript *per se* was elusive and post-transcriptional/splicing mechanisms regulating CSR have been understudied. To address if the sterile switch transcripts have a role in CSR, a mouse model of debranching enzyme (DBR1) haploinsufficiency and a knockdown approach in CH12F3A cells was used (15). These experiments revealed that if the generation of the transcript, post lariat debranching is abrogated, without affecting transcription or splicing, still, CSR is substantially impaired. The defect in CSR in DBR knockdown cells could be fully rescued by providing the switch RNA *in trans*, buttressing specificity of the phenotype (15). The defect in CSR was attributable to a failure to recruit AID to S regions, suggesting that the RNA might act *in trans* to guide AID back to the DNA locus due to sequence complementarity. This was indeed true, because provision of Sα transcripts *in trans* (in DBR1 knockdown CH12F3A cells) although could rescue AID recruitment to Sα but failed to target AID to Sμ, and thus failed to rescue CSR.

**Figure 4:**
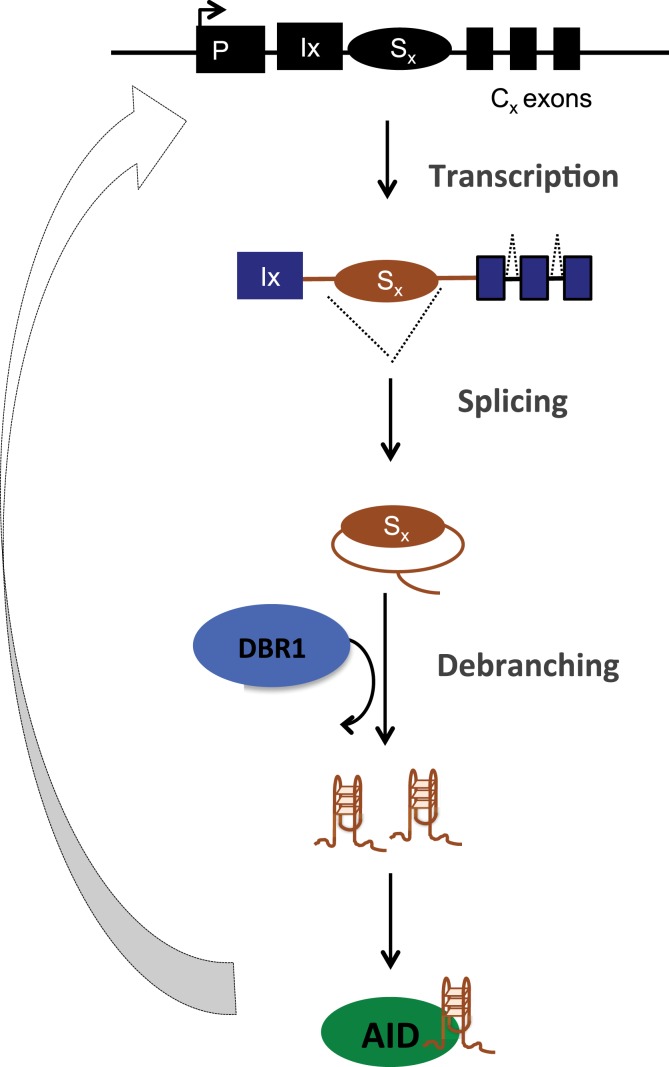
**Post-splicing RNA-dependent targeting mechanism of AID during CSR**. Transcribed S region are spliced, debranched (by DBR1) and the switch RNA assumes G-quadruplex conformation that allows structure- and sequence-dependent AID targeting back to S region DNA

The structural basis of AID binding to RNA was due to the ability of the S region RNA to fold into G-quadruplex structures, which could be recognized by a recognition motif in AID that has a conserved G133 residue (15). Interestingly, this residue was also mutated (G133V) in human patients with hyper IgM syndrome (57). Indeed, G133V AID could not be recruited to S regions in B cells and failed completely to rescue CSR in AID-deficient cells, despite being catalytically active, expressed at similar levels and having similar subcellular localization as WT AID (15). The model proposed based on these findings is that following transcription of the S regions, the spliced out lariat is debranched by DBR1, the debranched RNA assumes G-quadruplex structures, which facilitates AID binding and allows sequence- and structure-specific recruitment of AID to DNA during CSR (Figure 4). This physiological targeting mechanism can have implications for off-target AID activity as well because primary transcripts of many off-target AID genes (compared to non-target ones) have the potential to form G-quadruplex structures (15).

This opens up the field with many questions: how does the hand-off of AID-RNA complex to the DNA take place, and how the co-transcriptional mechanisms of AID targeting are complemented by this post-splicing mechanism to ensure a coordinated system enabling efficient CSR.

## Epigenetic Control of DNA Repair

The culmination of AID activity and lesion processing at recombining S regions during CSR is the generation of DNA DSBs, which constitute one of the most toxic lesions in a cell. Every cell in the body has evolved highly efficient and elaborate system dedicated to repair DSBs in order to promote survival and prevent translocations. However, a B cell faces a daunting challenge; not only does it have to repair the breaks to limit translocation potential, but it also has to time the process with such exquisite precision that it promotes long-range recombination. The non-homologous end-joining repair pathway is the most well-characterized one that is used during CSR (10). However, other pathways, such as, homologous recombination and alternative end-joining also play a role albeit to a lesser extent (8). The function of major players (genetic) in the AID-induced DNA damage response/repair phase that allow recombination including ATM, H2AX, Ligase IV, 53BP1, and Rif1 have been studied in-depth and reviewed elsewhere in Ref. (8, 10, 11, 13).

DNA insults have to be dealt within the context of a highly organized chromatin microenvironment and it is rational to assume that DNA DSB repair would be modulated significantly by epigenetic factors (58–60). Histones and many other DNA repair proteins undergo different post-translational modifications (PTMs) including phosphorylation, ubiquitylation, acetylation, methylation, sumoylation, and PARylation during an active repair response (61). These modifications can serve multiple purposes including serving as scaffold to recruit essential DNA repair factors, many of which harbor complementary domains to recognize the PTM. The histone variant H2AX gets phosphorylated at Ser 139 (γH2AX) in response to DNA damage by PI3K-like family of kinases (ATM, ATR, and DNA-PKcs), a classical DNA DSB marker. H2AX deficiency compromises CSR possibly due to defect in long-range chromatin remodeling and synapsis. BRCT domain containing proteins like MDC1 and 53BP1 also play an important role in the cascade of DNA repair events following DSB induction (62). MDC1, via its BRCT domain recognizes γH2AX and allows recruitment of E3 ubiquitin ligases RNF8 and RNF168, both of which are required for efficient CSR. The CSR defects are milder as compared to that in 53BP1-deficient B cells (63–65). 53BP1 functions mainly to prevent resection of DNA ends by recruiting Rif1 (66), and allows for timely persistence of breaks to be joined *in trans* (to acceptor S regions) rather than in cis (intra-switch recombination). 53BP1 recruitment to DNA is dependent on H4K20me2 mark, which is recognized via its tudor domain. Mutation in the tudor domain leads to compromised CSR, implying that reading the chromatin mark is important in potentiating its function (67). 53BP1 has been recently shown to recognize DNA damage-induced H2AK15 ubiquitylation (68), and it remains to be seen if this function also regulates CSR. The histone methyl transferase MMSET (WHSC1, implicated in Wolf-Hirschornn Syndrome) also functions during CSR, since knockdown of MMSET impairs 53BP1 recruitment to DNA damage site and compromises CSR (69). Lastly, the bromo-domain reader, Brd4, was also shown to function during CSR by providing an ideal chromatin platform during DNA repair phase (70). Brd4 inhibitor JQ1 perturbed CSR by affecting 53BP1 accumulation and end-joining pathway choice (70). JQ1 also compromised *in vivo* GC response during T-dependent Ag challenge, and this was suggested to be due to failure of NF-kappa B signaling and Bcl6 upregulation (71). However JQ1 can have non-specific effects, and genetic or domain-specific mutational approaches to test Brd4 function must be employed in the future to establish its role in CSR.

The methyl cytosine dioxygenase family of enzymes (TETs), has been in focus for their ability to demethylate specific regions of the genome (indirectly, via conversion of methylated cytidines to hydroxymethylated cytidines followed by engagement of base-excison repair) derepress genes (2). These TET enzymes have been found to be mutated or shut-off in many tumors, suggesting that they might have epigenetic tumor suppressive functions. Indeed, deletion of TET1 in mice was associated with increased propensity of development of B cell lymphomas (72). TET1 was required for maintenance of normal 5hmC levels and distribution across the genome, which allowed adequate expression of essential DNA repair genes (e.g., *lig1*, *ogg1*, *rad50*, and *rad51*). Absence of TET1 led to increased γH2AX foci and DNA damage sensitivity due to lack of repair and potentiated lymphomagenesis (pre-malignant B lymphomas at pro-B cell stage) (72). Outstanding questions about the role of other TET enzymes (TET2 and TET3), and even AID as a demethylase in B cell physiology and pathology remain to be explored (73, 74).

Lastly, chromatin remodeling complexes too have been implicated in reorganizing the chromatin during CSR. Defects in INO80 nucleosome remodeler in humans and in CH12F3A cells compromises CSR, likely due to improper loading of the cohesion complex and synaptic complex formation (75). Indeed, defects in cohesion loading proteins do cause abrogation of CSR (76, 77). These findings need to be introspected further because INO80 and cohesion complex have global cellular functions that extend beyond CSR.

## Summary and Perspective

Taken together, a bevy of epigenetic factors including “histone codes”, ncRNA, micro-RNAs, and super-enhancers communicate and coordinate to provide a dynamic chromatin landscape, which is geared for optimal diversification of the antibody repertoire.

Advances in sequencing-based techniques and data from the ENCODE project have greatly propelled research in the last decade (78). B cell biology, especially CSR has been investigated at depths like never before, and this has revealed the complexities that underlie this mechanistically counterintuitive process, which the B cell has to accommodate as a cost for co-evolution with pathogens. The smooth collisions of the chromatin and enhancer landscapes that facilitate CSR have been unraveled, however a multitude of questions lie ahead.

New modifications of histones continue to be identified. For example, crotonylation (79), which is thought to be similar to acetylation in being catalyzed by p300 in presence of intracellular crotonyl-CoA (79, 80), and even its erasers have been identified (81). Given that histone acetylation has functions in CSR, it remains to be seen if and how crotonylation can modulate CSR. DNA methylation has been extensively studied over the years and its role in locus accessibility and gene expression (epigenomic) is well characterized. But recently there has been identification of RNA methylation (N^6^-methyl-adenosine) as an epitranscriptomic modification (1). Transcriptome-wide m^6^A-mapping has provided great insight into the prevalence and relevance of this RNA modification, and how it impacts gene expression (82). Identification of m^6^A “readers”, “writers” and “erasers” has also propelled epitranscriptomic research (82). Given that CSR is impacted significantly by an integral non-coding RNA (eRNA, seRNA, lncRNA, and micro-RNA) component, and that RNA methylation is more prominent in non-coding RNAs, it will be unsurprising if this modification impacts CSR (83–85). Additionally, a very recent identification of N^6^-methyl-deoxyadenosine modification in the DNA of lower eukaryotes as a new “epigenetic” mark also opens up the question whether the writers and erasers of this mark will impact CSR in higher eukaryotes (86–88).

Recent upsurge in research on the impact of microbiota on a multitude of processes in the immune system during health and disease has uncovered the significance of this symbiotic association over centuries of evolution (89). Given that microbes have been shown to produce metabolites that functionally impact the epigenetic status of the host (90), it is tempting to speculate that even antibody responses *in vivo* will be epigenetically shaped by commensal-derived metabolites.

Most research reviewed herein has focused on positive regulation of CSR. But, one aspect that has to be borne in mind is that *in vivo*, CSR is only one of the multiple cell-fates that a clonally expanding population of B cells undergoes. So, there has to be coordinated allocation to other cell-fates including plasma cell differentiation and memory B cell differentiation, possibly by negative modulation of CSR. There has to be active genetic and epigenetic modules that skew cell-fate decisions toward one versus another path. It is rational to presume that such fine-tuning would be necessary in order to achieve an overall humoral response encompassing all aspects of adaptive immunity (adaptability, specificity, and memory). Future research is warranted to understand molecular mechanisms of negative regulation of the individual modules of cell-fate decision programs in B cells during a GC response.

A programed DNA damage process, such as, CSR is rare and unique to B cells, the fundamental understanding of which will propel basic (understanding DNA repair, recombination, and immunity) and translational science (ontogeny of lymphomas and its therapies). Epigenetics undoubtedly has enormous influence on physiology (adaptive immunity) and pathology (autoimmunity and lymphomagenesis) (4). Thus, exciting times lie ahead for research in CSR due to the establishment of the solid framework of the epigenetic landscape governing CSR.

## Conflict of Interest Statement

The authors declare that the research was conducted in the absence of any commercial or financial relationships that could be construed as a potential conflict of interest.
